# Supplementary material for: Hidden in Success: Gendered Patterns of Suboptimal Care Engagement Among TB Patients Who “Successfully” Completed Treatment in South Africa
Source: medRxiv. 2025 Sep 7:2025.09.04.25335036. Preprint. [Version 1] doi: 10.1101/2025.09.04.25335036 (PMC12425060; doi:10.1101/2025.09.04.25335036)
Supplement: 1 [file NIHPP2025.09.04.25335036V1-supplement-1.pdf]

medRxiv preprint doi: https://doi.org/10.1101/2025.09.04.25335036; this version posted September 7, 2025. The copyright holder for this preprint (which was not certified by peer review) is the author/funder, who has granted medRxiv a license to display the preprint in perpetuity.
It is made available under a CC-BY 4.0 International license .

SUPPLEMENTAL TABLES

Table S1: Model Evaluation Metrics

Model	Number of Classes	AIC	BIC	Entropy
**Overall Cohort**				
Linear	2	29513.14	29547.59	0.001
Linear	3	29292.99	29340.36	0.496
Linear	4	29298.99	29359.28	0.402
Quadratic	2	26593.70	26641.07	0.948
Quadratic	3	**26298.14**	**26362.74**	0.940
Quadratic	4	26306.14	26387.96	0.573
Cubic	2	26467.96	26515.33	**0.955**
Cubic	3	26475.96	26540.56	0.478
Cubic	4	26322.81	26404.63	0.608
**Male Cohort**				
Linear	2	19976.90	20008.13	0.921
Linear	3	19982.90	20025.86	0.406
Linear	4	19988.90	20043.58	0.443
Quadratic	2	18403.11	18446.06	0.942
Quadratic	3	**18218.89**	**18277.47**	0.928
Quadratic	4	18226.89	18301.10	0.536
Cubic	2	18304.87	18347.83	**0.943**
Cubic	3	18221.66	18280.25	0.916
Cubic	4	18229.66	18303.87	0.552
**Female Cohort**				
Linear	2	9115.42	9141.01	**0.989**
Linear	3	9121.42	9156.61	0.398
Linear	4	9127.42	9172.20	0.747
Quadratic	2	**7720.05**	**7634.23**	0.958
Quadratic	3	7761.79	7809.77	0.977
Quadratic	4	7763.99	7724.77	0.959
Cubic	2	7877.12	7912.30	0.979
Cubic	3	7791.15	7839.13	0.966
Cubic	4	7799.15	7859.92	0.540

medRxiv preprint doi: https://doi.org/10.1101/2025.09.04.25335036; this version posted September 7, 2025. The copyright holder for this preprint (which was not certified by peer review) is the author/funder, who has granted medRxiv a license to display the preprint in perpetuity.
It is made available under a CC-BY 4.0 International license .

Table S2. Descriptive Statistics of Study Participant's Characteristics Stratified by Trajectory Group (All data are presented as N (%) unless stated otherwise)

Characteristic	Consistent (N=461; 84.1%)	Suboptimal After 2 Months (N=42; 7.7%)	Suboptimal from Initiation (N=45; 8.2%)
**Socio-Demographic Characteristics**			
**Gender**			
Male	306 (66.4)	27 (64.3)	34 (75.6)
Female	155 (33.6)	15 (35.7)	11 (24.4)
**Age [Median (IQR)]**	38 (30, 47)	41 (32, 57)	36 (31, 45)
**Relationship Status**			
Not in a relationship	310 (67.2)	30 (71.4)	35 (77.8)
In a relationship	151 (32.8)	12 (28.6)	10 (22.2)
**Level of Education**			
Primary and below	89 (19.3)	11 (26.2)	9 (20.0)
Grade 8 -11 (before Matric)	344 (74.6)	29 (69.0)	36 (80.0)
Matric and above	28 (6.1)	2 (4.8)	0 (0)
**Lives Alone**			
Yes	99 (21.5)	4 (9.5)	8 (17.8)
No	362 (78.5)	38 (90.5)	37 (82.2)
**Live with Children**			
No	88 (19.1)	9 (21.4)	6 (13.3)
Yes	275 (59.7)	30 (71.4)	31 (68.9)
Missing	98 (21.3)	3 (7.1)	8 (17.8)
**Employment Status**			
Employed	99 (21.5)	11 (26.2)	9 (20.0)
Unemployed	362 (78.5)	31 (73.8)	36 (80.0)
**Monthly Household Income (Rands)**			
<R2000	312 (67.7)	27 (64.3)	29 (64.4)
R2000 - R5000	113 (24.5)	13 (30.9)	13 (28.9)
>R5000	36 (7.8)	2 (4.8)	3 (6.7)
**Social Support**			
Low	21 (4.6)	2 (4.8)	1 (2.2)
Moderate	144 (31.2)	16 (38.1)	15 (33.3)
High	289 (62.7)	24 (57.1)	29 (64.4)
Missing	7 (1.5)	0 (0)	0 (0)
**Social Capital**			
Low	50 (10.8)	5 (11.9)	6 (13.3)
Medium	308 (66.8)	30 (71.4)	32 (71.1)
High	98 (21.3)	7 (16.7)	7 (15.6)
Missing	5 (1.1)	0 (0)	0 (0)
**Clinical Characteristics and Health Behaviors**			
**Ever had TB Before**			
Never	343 (74.4)	23 (54.8)	28 (62.2)
Yes, less than 2 years ago	30 (6.5)	11 (26.2)	9 (20.0)
Yes, more than 2 years ago	88 (19.1)	8 (19.0)	8 (17.8)
**HIV Status**			
Positive	219 (47.5)	18 (42.9)	16 (35.6)
Negative	221 (47.9)	22 (52.4)	27 (60.0)
Unknown	21 (4.6)	2 (4.8)	2 (4.4)
**Depression (PHQ-9)**			
None/Minimal	203 (44.0)	21 (50.0)	19 (42.2)
Mild	71 (15.4)	9 (21.4)	7 (15.6)
Moderate	135 (29.3)	10 (23.8)	15 (33.3)
Moderate-Severe	47 (10.2)	2 (4.8)	4 (8.9)
Missing	5 (1.1)	0 (0)	0 (0)
**Anxiety (GAD-7)**			
Minimal	286 (62.0)	24 (57.1)	28 (62.2)
Mild	115 (24.9)	15 (35.7)	12 (26.7)
Moderate	37 (8.0)	2 (4.8)	4 (8.9)
Severe	18 (3.9)	1 (2.4)	1 (2.2)
Missing	5 (1.1)	0 (0)	0 (0)
**Alcohol Use (AUDIT)**			

medRxiv preprint doi: https://doi.org/10.1101/2025.09.04.25335036; this version posted September 7, 2025. The copyright holder for this preprint (which was not certified by peer review) is the author/funder, who has granted medRxiv a license to display the preprint in perpetuity.
It is made available under a CC-BY 4.0 International license .

Low	402 (87)	36 (85.7)	38 (84.4)
Medium	34 (7.4)	5 (11.9)	6 (13.3)
High	9 (2.0)	1 (2.4)	1 (2.2)
Alcohol Dependent	9 (2.0)	0 (0)	0 (0)
Missing	7 (1.5)	0 (0)	0 (0)
**Knowledge, Attitudes and Beliefs**			
**TB Knowledge**			
Low	215 (46.6)	20 (47.6)	22 (48.9)
High	241 (52.3)	22 (52.4)	23 (51.1)
Missing	5 (1.1)	0 (0)	0 (0)
**HIV Stigma [Median (IQR)]**	16 (11, 20)	16 (11, 20)	14 (12, 18)
**TB Stigma [Median (IQR)]**			
Isolation	6 (4, 8)	6 (4, 8)	6 (4, 8)
Disclosure	8 (5, 10)	8 (5, 10)	8 (5, 10)
**Medical Mistrust**			
Low	130 (28.2)	10 (23.8)	12 (26.7)
Medium	173 (37.5)	15 (35.7)	21 (46.7)
High	118 (25.6)	12 (28.6)	11 (24.,4)
Missing	40 (8.7)	5 (11.7)	1 (2.2)

medRxiv preprint doi: https://doi.org/10.1101/2025.09.04.25335036; this version posted September 7, 2025. The copyright holder for this preprint (which was not certified by peer review) is the author/funder, who has granted medRxiv a license to display the preprint in perpetuity.
It is made available under a CC-BY 4.0 International license .

Table S3. Characteristics of Male Study Participants Stratified by Trajectory Group (All data are presented as N (%) unless stated otherwise)

Characteristics	Consistent (N=305; 83.1%)	Suboptimal After 2 Months (N=27; 7.4%)	Suboptimal From Onset (N=35; 9.5%)
**Socio-Demographic Characteristics**			
**Age**	39 (31, 47)	41 (32, 47)	36 (31, 49)
**Relationship Status**			
Not in a relationship	203 (66.6)	21 (77.8)	26 (74.3)
In a relationship	102 (33.4)	6 (22.2)	9 (25.7)
**Level of Education**			
Primary and below	71 (23.2)	5 (18.5)	6 (17.1)
Grade 8-11 (before Matric)	220 (72.1)	21 (77.8)	29 (82.9)
Matric and above	14 (4.6)	1 (3.7)	0 (0)
**Lives Alone**			
Yes	75 (24.6)	4 (14.8)	6 (17.1)
No	230 (75.4)	23 (85.2)	29 (82.9)
**Live with Children**			
No	71 (23.3)	6 (22.2)	5 (14.3)
Yes	160 (52.5)	18 (66.7)	24 (68.6)
Missing	74 (24.2)	3 (11.1)	6 (17.1)
**Employment Status**			
Employed	69 (22.7)	7 (25.9)	8 (22.9)
Unemployed	236 (77.3)	20 (74.1)	27 (77.1)
**Household Income in Rands**			
<R2000	210 (68.9)	19 (70.4)	22 (63)
R2000 - R5000	70 (22.9)	6 (22.2)	11 (31)
>R5000	25 (8.2)	2 (7.4)	2 (5.7)
**Social Support**			
Low	14 (4.6)	2 (7.4)	0 (0)
Moderate	99 (32.5)	11 (40.7)	12 (34.3)
High	187 (61.3)	14 (51.9)	23 (65.7)
Missing	5 (1.6)	0 (0)	0 (0)
**Social Capital**			
Low	32 (10.5)	2 (7.4)	4 (11.4)
Medium	197 (64.6)	20 (74.1)	27 (77.1)
High	71 (23.3)	5 (18.5)	4 (11.4)
Missing	5 (1.6)	0 (0)	0 (0)
**Clinical Characteristics and Health Behaviors**			
**Ever had TB Before**			
Never	222 (72.8)	12 (44.4)	24 (68.6)
Yes, less than 2 years ago	24 (7.9)	9 (33.3)	7 (20.0)
Yes, more than 2 years ago	59 (19.3)	6 (22.2)	4 (11.4)
**HIV Status**			
Positive	122 (40.0)	10 (37.0)	7 (20.0)
Negative	169 (55.4)	15 (55.6)	26 (74.3)
Unknown	14 (4.6)	2 (7.4)	2 (5.7)
**Depression (PHQ-9)**			
None/Minimal	141 (46.2)	13 (48.1)	16 (45.7)
Mild	44 (14.4)	6 (22.2)	7 (20)
Moderate	85 (27.9)	6 (22.2)	11 (31.4)
Moderate-Severe	30 (9.8)	2 (7.4)	1 (2.9)
Missing	5 (1.6)	0 (0)	0 (0)
**Anxiety (GAD-7)**			
Minimal	192 (63.0)	17 (63.0)	25 (71.4)
Mild	77 (25.2)	9 (33.3)	8 (22.9)
Moderate	22 (7.2)	1 (3.7)	1 (2.9)
Severe	9 (3.0)	0 (0)	1 (2.9)
Missing	5 (1.6)	0 (0)	0 (0)
**Alcohol Use (AUDIT)**			
Low	259 (84.9)	23 (85.2)	30 (85.7)
Medium	26 (8.5)	4 (14.8)	4 (11.4)
High	7 (2.3)	0 (0)	1 (2.9)

medRxiv preprint doi: https://doi.org/10.1101/2025.09.04.25335036; this version posted September 7, 2025. The copyright holder for this preprint (which was not certified by peer review) is the author/funder, who has granted medRxiv a license to display the preprint in perpetuity.
It is made available under a CC-BY 4.0 International license .

Alcohol Dependent	8 (2.6)	0 (0)	0 (0)
Missing	5 (1.6)	0 (0)	0 (0)
**Knowledge, Attitudes and Beliefs**			
**TB Knowledge**			
Low	132 (43.3)	13 (48.1)	17 (48.6)
High	168 (55.1)	14 (51.9)	18 (51.4)
Missing	5 (1.6)	0 (0)	0 (0)
**HIV Stigma [Median (IQR)]**	16 (11, 20)	14 (7, 17)	16 (12, 21)
**TB Stigma [Median (IQR)]**			
Isolation	6 (4, 8)	6 (4, 8)	6 (5, 8)
Disclosure	8 (5, 10)	8 (4, 10)	8 (6, 10)
**Medical Mistrust**			
Low	85 (27.9)	5 (18.5)	11 (31.4)
Medium	111 (36.4)	8 (29.6)	15 (42.9)
High	82 (26.8)	10 (37.0)	8 (22.9)
Missing	27 (8.9)	4 (14.8)	1 (2.9)

medRxiv preprint doi: https://doi.org/10.1101/2025.09.04.25335036; this version posted September 7, 2025. The copyright holder for this preprint (which was not certified by peer review) is the author/funder, who has granted medRxiv a license to display the preprint in perpetuity.
It is made available under a CC-BY 4.0 International license .

Table S4. Characteristics of Female Study Participants Stratified by Trajectory Group (All data are presented as N (%) unless stated otherwise)

Characteristic	Consistent (N=162; 89.5%)*	Suboptimal from Onset (N=19; 10.5%))
**Socio-Demographic Characteristics**		
**Age**	38 (30, 48)	30 (25, 42)
**Relationship status**		
Not in a relationship	129 (79.6)	13 (68.4)
In a relationship	63 (38.9)	6 (31.6)
**Level of Education**		
Primary and below	22 (13.6)	5 (26.3)
Grade 8 -11 (before Matric)	126 (77.8)	13 (68.4)
Matric and above	14 (8.6)	1 (5.3)
**Lives Alone**		
Yes	23 (14.2)	3 (15.8)
No	139 (85.8)	16 (84.2)
**Live with Children**		
No	20 (12.3)	1 (5.3)
Yes	119 (73.5)	15 (78.9)
Missing	23 (14.2)	3 (15.8)
**Employment Status**		
Employed	32 (19.8)	3 (15.8)
Unemployed	130 (80.2)	16 (84.2)
**Monthly Household Income (Rands)**		
<R2000	105 (64.8)	12 (63.2)
R2000 - R5000	46 (28.4)	6 (31.6)
>R5000	11 (6.8)	1 (5.3)
**Social Support**		
Low	7 (4.3)	1 (5.3)
Moderate	47 (29.0)	6 (31.6)
High	106 (65.4)	12 (63.2)
Missing	2 (1.2)	0 (0)
**Social Capital**		
Low	19 (11.7)	4 (21.1)
Medium	114 (69.5)	12 (63.2)
High	29 (17.9)	3 (15.8)
**Clinical Characteristics and Health Behaviors**		
**Ever Had TB Before**		
Never	126 (77.8)	10 (52.6)
Yes, less than 2 years ago	7 (4.3)	3 (15.8)
Yes, more than 2 years ago	29 (17.9)	6 (31.6)
**HIV Status**		
Positive	99 (61.1)	15 (78.9)
Negative	57 (35.2)	3 (15.8)
Unknown	6 (3.7)	1 (5.3)
**Depression (PHQ-9)**		
None/Minimal	67 (41.4)	6 (31.8)
Mild	27 (16.7)	3 (15.8)
Moderate	51 (31.5)	7 (36.8)
Moderate-Severe	17 (10.5)	3 (15.8)
**Anxiety (GAD-7)**		
Minimal	96 (59.2)	8 (42.1)
Mild	40 (24.7)	8 (42.1)
Moderate	16 (9.9)	3 (15.8)
Severe	10 (6.2)	0 (0)
**Alcohol Use (AUDIT)**		
Low	149 (92.0)	15 (78.9)
Medium	8 (4.9)	3 (15.8)
High	3 (1.9)	0 (0)
Alcohol Dependent	0 (0)	1 (5.3)
**Knowledge, Attitudes and Beliefs**		
**TB Knowledge**		
Low	86 (53.1)	9 (47.4)

medRxiv preprint doi: https://doi.org/10.1101/2025.09.04.25335036; this version posted September 7, 2025. The copyright holder for this preprint (which was not certified by peer review) is the author/funder, who has granted medRxiv a license to display the preprint in perpetuity.
It is made available under a CC-BY 4.0 International license .

High	76 (46.9)	10 (52.6)
**HIV Stigma [Median (IQR)]**	16 (12, 21)	20 (12, 23)
**TB Stigma [Median (IQR)]**		
Isolation	6 (4, 8)	7 (4, 8)
Disclosure	9 (5, 10)	10 (7, 11)
**Medical Mistrust**		
Low	46 (28.4)	5 (26.3)
Medium	65 (40.1)	10 (52.6)
High	37 (22.8)	4 (21.5)
Missing	14 (8.6)	0 (0)
